# Patient perceptions and preferences during a community-based telehealth care model for moderate-to-severe hypertension in rural communities in Kenya and Uganda

**DOI:** 10.1371/journal.pone.0350915

**Published:** 2026-07-28

**Authors:** Jason Johnson-Peretz, Andrew Mutabazi, Ambrose Byamukama, Lawrence Owino, Cecilia Akatukwasa, Anjeline Onyango, Fredrick Atwine, Titus O. Arunga, Sabina Ogachi, Asiphas Owaraganise, Norton Sang, Erick M. Wafula, Nicole Sutter, Colette Aoko, Jane Kabami, Elijah Kakande, Gabriel Chamie, Laura B. Balzer, Maya L. Petersen, Moses R. Kamya, Diane V. Havlir, Carol S. Camlin, Matthew D. Hickey, James Ayieko

**Affiliations:** 1 University of California, San Francisco (UCSF), Division of Prevention Sciences, San Francisco, California, United States of America; 2 Infectious Diseases Research Collaboration, Kampala, Uganda; 3 Kenya Medical Research Institute (KEMRI), Kisumu, Kenya; 4 University of California, San Francisco (UCSF), Dept of Medicine, San Francisco, California, United States of America; 5 University of California, Berkeley, Divisions of Biostatistics, Epidemiology, and Computational Precision Health, Berkeley, California, United States of America; 6 Department of Medicine, Makerere University College of Health Sciences, Kampala, Uganda; Aga Khan University, PAKISTAN

## Abstract

**Introduction:**

Hypertension is a growing health concern in sub-Saharan Africa, yet access to care can be challenging, especially in rural areas. Overcoming barriers to hypertension care remains a priority. Pairing community health worker (CHW) and telehealth modalities offers one way to extend the reach of hypertension care without overburdening patients or healthcare systems in rural areas.

**Methods:**

This qualitative study was nested within the SEARCH Sapphire pilot randomised controlled trial testing hypertension treatment through CHW-facilitated, clinician-driven telehealth (intervention) compared to clinic-based care (control) for adults aged ≥40 years with moderate-severe hypertension in rural western Kenya and southwestern Uganda. We conducted audio-recorded, in-depth, semi-structured interviews with purposively selected healthcare providers (clinicians and CHWs; N = 15) and participants (N = 40) between January-April 2023 to explore hypertension diagnosis and linkage to care; experiences with community hypertension care; family and work contexts; and the integration of telehealth into clinic and CHW workflows.

**Results:**

Overall, participants felt satisfied with community-delivered telehealth care for hypertension. Participants noted community-based care saved on transport costs and reached those who were unwell or who lived far from clinic. Intervention arm participants felt CHWs were suitable for routine hypertension care, worked closely with clinicians, and could increase health literacy within the community. Participants desired a model with clear communication and involvement of the facility-based clinician, CHW, and participant. Providers found telehealth to be of similar quality to clinic-based care for routine hypertension treatment, though noted that clinic-based care is at times needed for more comprehensive clinical evaluation or to provide additional healthcare services.

**Conclusions:**

Participants and providers indicated overall positive attitudes and receptivity to CHW-facilitated telehealth for hypertension care. CHW-delivered telehealth for community-based hypertension care offers one way to improve hypertension treatment outcomes in a manner that prioritizes patient-centeredness and maintains care quality.

**Trial Registration:**

NCT04810650 Registered on 2021-03-18.

## Background

In an era of simultaneous health system strengthening and funding constraints, new ways to extend the efficiency and reach of clinical care for chronic diseases without compromising quality of care are paramount. One such chronic disease is hypertension (HTN). HTN is highly prevalent in southern, central, and eastern Africa, with a growing burden in rural areas [1,2]. Treatment for HTN in this region is low, despite being a leading contributor to chronic kidney disease and cardiovascular disease (CVD) associated deaths [3,4]. Only about a quarter of the population with HTN know their diagnosis, and as few as 16% of people have achieved HTN control [1,5]. Population-level screening and linkage to clinic-based treatment can improve outcomes, though benefits are limited by challenges retaining patients in clinic-based care due to congested clinics, long travel times, and opportunity costs of frequent clinic appointments [6–8]. In low- and middle-income countries, the use of community health workers (CHWs) and telehealth visits to broadly screen for and monitor chronic conditions shows promise as two such means [9,10]. Telehealth integration may potentiate CHW-delivered community-based HTN care.

We define telehealth as the use of mobile and digital technologies to facilitate care delivery and improve health. Telehealth can help overcome transportation barriers, including for people in rural areas who reside far from the nearest clinic [3]. Despite wide uptake across Africa since the COVID-19 pandemic, multiple barriers limit telehealth’s full potential [11]. A key barrier is a general lack of awareness about telehealth. Other barriers include inconsistent implementation policies, limited adoption of electronic medical records, language challenges in platform development, and unclear funding or reimbursement [12]. Additionally, chronic disease management by telehealth varies by condition, influenced both by social perceptions of care seeking (e.g., stigma around mental health) and requirements for in-person procedures (e.g., blood draws) [13]. The Africa Telehealth Collaboration (ATC) has proposed four focus areas to address these barriers: education and training for healthcare professionals; infrastructure requirements; regulatory requirements and industry engagement; and user perspective and concerns (both patient and practitioner) [14]. With regard to the last focus area – the need to gain trust and communicate the availability of telehealth options to different communities – pairing telehealth with CHW home visits offers a potential solution [3].

For HTN specifically, studies have demonstrated that CHWs are effective at improving HTN and CVD risk screening, measuring blood pressure in community settings, encouraging medication adherence, and linking patients to care [15–20]. Strategies to bring hypertension treatment into the community can further improve blood pressure control compared to clinic-based care [21]. Algorithms deployed within phone-accessible platforms or apps can help CHWs with HTN diagnosis [22]. Additionally, mobile phones have the capacity to extend certain kinds of specialised care from a central clinic to surrounding rural areas in a hub-spoke-like fashion, enhancing timely availability of expertise to frontline providers [23]. CHWs are an effective means of connecting the hub to its community-based endpoints, thereby overcoming patient-level barriers like transportation costs; CHWs are the ‘spokes’ of the wheel [10]. Nonetheless, both training in local languages to facilitate the process and supervision and the auditing of CHW knowledge are also essential for maintaining standard of care [21,22].

Despite the broad rollout of telehealth in Africa and research on its spread, not much is known about patient perceptions of telehealth for managing hypertension, especially when facilitated by CHWs. As Donelan et al. note, “Understanding the perceived relative value of different modes of healthcare services may help to shape the use of virtual or remote healthcare technologies” [24]. One priority, therefore, is understanding how patients perceive clinic-based hypertension care models compared to telehealth-oriented, community-based care; and, secondarily, to round out the picture of telehealth acceptability within the clinic-to-consumer service pathway, integrating clinician and CHWs perceptions about telehealth for HTN care. To answer these questions, we embedded a qualitative sub-study within a pilot randomized controlled trial conducted as part of the SEARCH Sapphire study (NCT:04810650) testing a clinician-driven, CHW-facilitated telehealth intervention to improve hypertension control among adults with moderate-severe hypertension in rural Uganda and Kenya. We sought to assess people’s experiences with each model (community vs clinical care), including their preferences and perceptions about quality of care. From this comparison of models, we derived evidence for both the general acceptability of the telehealth model and participant-perceived limits to its utility (i.e., the range of acceptability).

## Methods

### Study design

The main study’s methods and quantitative findings have been published elsewhere (NCT04810650 2021-03-18) [25]. Briefly, after community-wide screening by CHWs conducted between 12 May and 1 November 2022, participants aged ≥40 years were eligible for the study if blood pressure was elevated at both community-based and clinic measurement (≥140 mmHg systolic or ≥90 mmHg diastolic) and moderately-severely elevated on at least one of these measurements (≥160 mmHg systolic or ≥100 mmHg diastolic). After an initial clinic visit for hypertension care, participants were randomly assigned to intervention or control arms [25]. Control participants received patient-centred follow-up care from a study clinician at the government-run primary health centre in their community. Intervention arm participants received follow-up care at home via a Ministry of Health (MoH) CHW home visit and phone-based telehealth with a study clinician based at the participant’s local community clinic. During CHW home visits, the CHW measured the participant’s blood pressure, conducted an adherence assessment with pill count, consulted the clinician via telephone, and, following the clinician’s direction, provided the participant with pre-packaged hypertension medication. The CHW recorded visit data on study-provided smartphones using Open Data Kit. During the phone consult, the clinician reviewed the CHW’s report, spoke with the participant, counselled them as necessary, and prescribed medication. The clinician recorded visit data on the paper-based MoH medical records and in a tablet-based case report form. CHWs received phone airtime and a stipend for their time. Throughout, we refer interchangeably to CHWs, community health promoters (CHP; Kenya) or village health team (VHT; Uganda). The intervention lasted 48 weeks. Loss to follow-up was minimal, though slightly higher in the control (clinic-based) arm (3 vs 9 participants).

### Qualitative data collection procedures

For the qualitative sub-study, a gender-balanced team (3 men, 2 women) of trained qualitative researchers conducted audio-recorded, in-depth, semi-structured interviews with purposively selected providers (N = 15) and participants (N = 40) from the main trial between 25 January and 28 April 2023 (Table 1); participants were balanced by sex, community, blood pressure status at 6 months (elevated or controlled), and HIV status, but selection was agnostic to intervention satisfaction as our intention was to uncover degrees of satisfaction through a random selection within these purposive categories. Clinicians were all study staff. We achieved data saturation with these numbers of participants.

**Table 1:** Participant demographics.

Participants	Control (n = 18)	Intervention (n = 22)
Age, mean (min, max)	58.6 (48, 75)	59.3 (41, 77)
***Sex***		
Female	10	13
Male	8	9
***Country***		
Kenya	8	10
Uganda	10	12

Providers	(n = 15)	
Age, mean (min, max)	42 (29, 62)	
***Role***		
CHW	10	
Clinicians/Clinic Team	5	
***Sex***		
Female	6	
Male	9	
***Country***		
Kenya	8	
Uganda	7	

The interviewers, who were native speakers of the local languages (English, Luo, Runyankole, and Swahili), consented participants and conducted interviews in locations convenient for the participants to ensure confidentiality. Patient interviews explored hypertension diagnosis experience, enrolment and linkage to care; experience with community hypertension care, beliefs about hypertension, including how it compares to other chronic-managed illnesses like HIV and diabetes; family and work contexts, including capacity to make lifestyle changes; and medication adherence motivations, challenges and facilitator strategies. Provider (CHW, clinician, and research assistant) interviews explored clinic capacity and workflow for addressing patients with hypertension and integration of telehealth into the CHW workload; views on the importance of treating hypertension and its impact on patients; confidence in screening and diagnosis skills; and experience with clinic- and community-based care, including who benefits from each. From both cohorts, we solicited recommendations to improve hypertension care. In line with the qualitative tradition, we used open-ended questions to elicit feelings, beliefs, and perceptions about the intervention (whether positive or negative), and a semi-structured format to delve into the reasons for those feelings. This allowed us to uncover a broad range of information not captured in quantitatively-focused surveys that deploy *a priori* conceptual categories of acceptance or rejection of an intervention component. We have included the interview guides as supplementary material for reference; the pertinent questions appear in Table 2.

**Table 2:** Question stems concerning experiences with and acceptability of hypertension care.

*Patient-directed question stems*
What do you think about the idea of community-based care for high blood pressure? a) In what situations is it better to get care at the clinic? b) In what situations is it better to get care at home?
How does community-based care for high blood pressure make it easier to receive high blood pressure treatment?
How does community-based care for high blood pressure make it harder to receive high blood pressure treatment?
What concerns do you have about community-based care for high blood pressure?
***Community Health Worker directed question stems***
What do you think about the idea of community-based hypertension care? Tell me about the patients for whom you think it was useful. 1. Probe: Can you tell me a story about one of them? 2. Tell me about the patients for whom it was not useful or did not work well. 3. Probe: Can you tell me a story about one of them?
What issues have you experienced with contacting clinicians to conduct phone-based hypertension care assessment? Are they ever not available and, if so, what do you do?
***Clinician-directed question stems***
What do you think about the idea of community-based hypertension care? Tell me about the patients for whom you think it was useful. Tell me about the patients for whom it was not useful or did not work well.
How do phone visits for community-based hypertension care fit into your workflow? What are some of the challenges you have experienced?
Where there times where you weren’t able to get enough clinical information during a phone visit or where you had to ask a participant to come to the clinic? Tell me about those experiences. What would have made these experiences easier?
***Question stems for both CHWs and Clinicians***
How does the quality of care differ between clinic-based and community-based (with clinician phone consultation) hypertension care?
What concerns do you have about community-based hypertension care?
What elements of community-based hypertension care do you think were most impactful on the trial participants?

### Analytical approach

Team members transcribed and translated the audio recordings into English. A six-person team coded data using Dedoose software and a codebook developed using the R-EIGHT method to create code families accounting for both *a priori* and inductively derived research foci [26]. We conducted a two-phase analysis: first, a thematic analysis based on priority questions exclusive of providers, followed by a closer framework analysis inclusive of providers*.* The thematic analysis looked at excerpts from the following eight codes: Experiences with community HTN care; Clinic visit experiences and home visits; Linkage to care and clinic experience, including conversations with provider, linkage experience, and feelings around being in an HIV-HTN combined clinic, and finally, medication access challenges. The framework analysis, in contrast, focused on excerpts associated with four additional codes and keywords: Phone visits, phone (keyword), general thoughts, and CHWs. Together, these analyses resulted in analytic memos across several broad and emergent themes.

### Ethical approval and informed consent

The institutional review boards of the Kenya Medical Research Institute (KEMRI) in Kenya; the Infectious Disease Research Collaboration (IDRC) in Uganda; and the University of California San Francisco (UCSF) in the United States of America all granted ethical approval to this study, which is registered on ClinicalTrials.gov (NCT04810650). All participants provided written informed consent. The UCSF ethical review board considers a range of ethical guidelines: https://irb.ucsf.edu/regulations-statutes-guidance. Specifically, the following codes/ ethical standards were used by the UCSF IRB in review of this study: The Belmont Report, Office of Human Research Protections (OHRP): Protection of Human Subjects 45 CFR 46.

## Results

Overall, participants who were exposed to CHW-facilitated telehealth (those randomised to the intervention arm) felt a high degree of satisfaction with the intervention. In contrast, those in the control arm of the study who received hypertension care at the clinic had some reservations about the *idea* of community-based care, including potential concerns about the quality of care and misconceptions that CHW telehealth would preclude them from receiving clinic-based care. *Participants in both arms, however,* expressed consensus that community-based care saved or would save on transport costs and reach those who live at a distance from public transport routes. Additionally, participants thought that receiving medication at home could also help those who were old or unwell because of physical injury or an acute illness and could not easily attend facilities. With only modest differences, these attitudes held when compared by country, sex, and age, though Ugandan participants expressed somewhat more preference for facility-based care. More notable differences became apparent when we compared control and intervention participants as described further below.

Below, we examine participant and provider perceptions of CHW-delivered telehealth visits compared to clinic-based care. We begin with a brief overview of the forms of sociality that potentially influenced reception of telehealth and community care before reporting attitudes common to participants in each arm of the study. We then describe control participant perceptions before examining how the intervention interfaced with these basic attitudes. We close with an examination of provider views, drawn from both clinicians and CHWs.

### Personal relationships with providers

Participants emphasized the importance of personal relationships with providers for building trust and the important role of social connection in promoting engagement in care:

“Some clients are used to interacting or talking to the first person they met on day one when they came to the facility. It means, therefore, that they will start making a lot of enquiries if you happen to be a new staff member. When you are making calls, you have to introduce yourself to the participant. It is a challenge because some people would want to be served by one person year in, year out. Some clients will question if they are called by different people. However, with the rapport that we have created with these people, we have managed to reduce that mentality.” (36 y.o. male Research Assistant, Kenya)

This extended to CHWs as well. One CHW explained the importance of direct three-way communication between healthcare providers, CHWs, and patients during CHW home visits for hypertension care where clinicians were consulted via telehealth. This CHW explained, “*When the client hears what the doctor has said, the client is encouraged and knows that what the VHT said is right, rather than <a CHW> getting the <blood pressure> readings then consulting the doctor later*.” (55 y.o. female CHW, Uganda). Once the participants understood the social network that bound a specific CHW to a specific clinician and to the participant him- or herself, they were more open to receiving community care. As one intervention arm participant, who felt that community care could reach those who did not recognize that their symptoms could be treated, explained,

“<Community care> is a good idea, and it makes it cheap for us to access high blood pressure care and treatment. At the same time, this community-based care for high blood pressure would increase awareness creation. When providers come to my place and they find me with other people, those people will ask me who you are. Of course, I will tell them, and from there, some may ask me again what the signs of high blood pressure are. They will also develop that interest to know their status concerning High blood pressure.” *(57 y.o. Male, intervention arm, Kenya)*

For similar social reasons, when comparing facility-based care with community-based care, participants noted that facilities provided comfort for participants by letting them see they were not the only ones with a hypertension diagnosis. At a clinic, participants could interact with other patients in hypertension care and learn from them.

“At the clinic, one interacts with fellow patients, which provides courage on the grounds that I am not the only sick person in the world. People may be suffering from different ailments, but all in all, I am not the only sick person, including those who may be sharing the same condition as mine.” (70 y.o. male, intervention arm, Kenya)

### Control arm speculations on community-based care

*Control arm* participants received hypertension care only in the clinic and did not experience the CHW telehealth intervention. They offered their perspectives about community-based care based either on their own perceptions of CHWs or, as above, on what their neighbours in the intervention arm said about the study. A few control participants operated under the misconception that community-based care would preclude clinic visits. “*Patients cannot receive hypertension care from the community with ease because they do not have a chance to interact with their providers*.” (53 y.o. female, control arm, Uganda) They also questioned the quality of community care, which they saw as provided exclusively by CHWs. One stated, “*I do not trust the CHWs, they don’t seem to keep patients’ information confidential, and they are not professionals. So, I generally do not like being served by CHWs*.” (63 y.o. male, control arm, Uganda) Other control participants raised concerns that CHWs do not carry the full range of medications with them, were busy juggling multiple responsibilities, were unreliable, or may set up their own business with the medication.

“I prefer being seen at the facility because there are some medications that if the CHV is given, they will start a business. I might be sick and come tell the provider that I am sick. They would test me and then tell me, ‘*We have run out of medication at the clinic, but you can buy them at this place. Do you have the money?*’ and I do not have the money. If I came here <to the clinic>, he would not let me go back empty-handed; he would give me some and tell me to go with this and come back on such a day.” (55 y.o. female, control arm, Kenya)

In case of a medication stock-out at the local clinic, a facility trip meant the chance to go to a private pharmacy, which might not be available within the person’s local village.

“At the clinic, the provider gives ample to time to discuss all your health issues… and I might find the facility has no drugs, so I can use the chance to buy from private clinics.” (61 y.o. male, control arm, Uganda)

Control arm participants tended to feel that facility-based care offered more benefits than community-based care, including access to the most up-to-date medications and the ability to test for and treat other conditions. This perception appeared more pronounced in Uganda compared to Kenya. In their view, participants suggested facility-based care was better for comprehensive check-ups and changing medical prescriptions; participants felt they could change their regimen more easily at the clinic.

“It is good to receive your care at the clinic when there is need for your regimen to be changed. It would be easier to find the medication at the clinic than if they come to the community and forget to carry that medication with them. I think that will be the benefit of receiving care at the clinic. Community-based care, on the other hand, will be beneficial in terms of travelling cost.” (50 y.o. male, control arm, Kenya)

When control arm participants saw home-based care being provisioned by clinical providers, they had more favourable views of the quality of service. “*Home-based care also helps the provider to learn about* *one’s living situation and challenges one may be undergoing*.” (49 y.o. male, control arm, Kenya)*.* Other participants recognised that CHWs acted as go-betweens for the patient and clinician:

“When the CHV brings drugs, I think <the CHW> will be sent by the doctor after the doctor has prescribed those medications. They will have to bring me those medications and pass on the information that the doctor said. First, they will have to explain to me how I am going to take the medications. They will have also to enquire with me about my progress and give feedback to the doctor.” (52 y.o. female, control arm, Kenya)

For these participants, community care was preferred for routine care and medication refills, though one control participant conveyed challenges communicating with CHWs who also have other responsibilities.

“Those people back in the community have not benefited much, because we have heard them complaining about how they find it hard to access their providers. Sometimes VHT’s phones are never on, and some VHTs are always in their gardens, so you can’t have them in the time you need them. Generally, my fellow women are jealous of my delivery option; they wonder whether I had to bribe them to keep receiving care from the clinic. But I always explain to them how all that happened out of a raffle draw.” (53 y.o. female, control arm, Uganda)

Even so, control participants acknowledged that CHWs did not always do the best counselling when compared to providers at the clinic.

“I would have preferred the option of community, but already my diabetes medication is got from the clinic, and again you can’t get the best counselling from the community VHTs. … Receiving care from the community saves transport, but on the side of clinic, I will have got a chance to interact with the provider. You know counselling is more important than even the drugs.” (62 y.o. male, control arm, Uganda)

Of course, interpersonal politics and disagreements with a CHW also could affect people’s perceptions of this mode of service delivery:

“You know people in the community are very funny – there could be a VHT from a certain home that people don’t go along with, and someone would rather decide to put in transport to the facility to be worked on by a health provider.” (63 y.o. male, control arm, Uganda)

### Intervention arm experiences with community-based care

*Intervention arm participants* directly experienced community-based telehealth care for hypertension and had more favourable views of this modality of receiving care than control arm participants who received care in the clinic. Intervention arm participants recognized that receiving community-based care did not preclude going to the facility for additional care.

“Other than the transport issue, I find care in both places the same because you are given the same medication, whether in the community or at the clinic…. There’s no challenge with the community option because if you fail to have any service in the community, you are free to go to the clinic and get served from there.” (75 y.o. male, intervention arm, Uganda)

Another participant remarked, “*If they are using medication and feel that it is not helping, then they can go to the facility and get checked again, but if they do not have any challenge and it is brought to them, then they would just use it*.” (60 y.o. female, intervention arm, Kenya). They also recognized that CHWs were not the only ones to administer home-based telehealth care, even if CHWs were the primary medication couriers. Intervention arm participants also noted that CHWs did not draw blood and that they would need to go to the clinic for this service.

CHWs were seen as good for routine care, working closely with clinicians. As one participant phrased it, “*When all is well, there’s no need for going to the health facility*.” (42 y.o. male, intervention arm, Uganda). Another observed that,

“Many people come from far and since they do not feel like they are seriously sick, they might end up missing their appointment. If they have no money for transport, they may end up defaulting. When they asked us how to get a solution to this problem, personally I suggested that we have some community health volunteers (CHWs) in the community, and they work in the facility… Now I want to admit that this has been working well on my part; they bring me drugs at home.” (57 y.o. male, intervention arm, Kenya*)*

Intervention-arm participants trusted the training provided to CHWs. Even so, some participants preferred getting care where they get their medication – at the clinic due to the broader array of services offered at clinic – and some participants approved of both:

“If I knew some of its disadvantages, I would not be enjoying the CHW visit which I believe is key; if the CHW was not bringing the drugs in time, I would have complained. But so far, I think it is a good model and I see no situation when home-based care is not appropriate. … Clinic is also best because one is likely to meet a new idea or diagnosis, unlike the CHW, who is limited to certain tasks every now and then.” (56 y.o. female, intervention arm, Kenya*)*

On the other hand, intervention participants voiced a concern that clinic-based care often meant having to wait – and even if served quickly, they still would lose time that could have been spent working: “*When I* *come for appointments, I am forced to forego my work. Therefore, in case I spend a lot of time here at the facility, it means I will be losing a lot back at my workplace. Maybe I was given some hours to rush to the facility, and when I go back, it is past <the allotted> time already. Meaning you will have problems with your employers*.” (57 y.o. male, intervention arm, Kenya) Men especially expressed concern about missing time working because of facility-based appointments, but if the woman was the breadwinner, it was also her concern:

“You know, for me, I am the breadwinner of my family. I have work for food for my family, meaning that any time wasted while going to the clinic would cost me a lot.” (58 y.o. female, intervention arm, Uganda)

“My family members are happy that I <received CHW telehealth> because frequent travelling to the facility is hectic. In fact, on the scheduled date, I ensure I wait for the CHV till he is through with the services and that is when I can start my usual chores. He advises that I don’t do heavy tasks and, if best, I relax to the maximum till he takes the blood pressure readings. Usually, he gets through by either 9am or 10am.” (56 y.o. female, intervention arm, Kenya)

Home visits thus ensured greater tranquillity and privacy. Intervention arm participants also liked that CHWs could help navigate treatment changes and side effects. They also noticed when the CHWs did a pill count: “*Whenever they come, I give the remaining pills, and they top up new ones…I think this is their sure way of confirming that I am adhering well to my medication.”* (56 y.o. female, intervention arm, Kenya)

Intervention-arm participants mentioned that CHWs helped promote community literacy about different diseases. They took care to coordinate well with participants to find them in the community or deliver medication at home. Participants did note that CHWs being members of the community, however, had both benefits and drawbacks for treatment engagement.

“The health workers have been good … I am close to the VHT’s home, so I can walk there, but others are not all that, so I have heard some village members complain. Some say when the VHTs were given phones, they went to another economic class hence not minding <their VHT responsibilities>.” (52 y.o. female, intervention arm, Uganda)

“I am extremely happy about that <the CHW being a member of the community> and this is because this is a person who knows me better and is supposed to know me better, when I am down with illness, he is affected directly – and even if I die, he is directly affected, too! <laughter> It is therefore important that develop a positive attitude towards his services and vice versa.” (56 y.o, female, intervention arm, Kenya)

### Views from CHWs, clinicians, and clinical team members

*Clinical team members* found telehealth good for straightforward care; in-person clinics might be needed for some care that required a more detailed history or physical examination.

“There is not much difference, but looking at the phone consultation, there are things the clinician is not able to pick as compared to when the session is in-person. At the end of the day, the patient still has the opportunity to explain every aspect of their concerns via phone. The BP measuring process is nothing to worry about because the CHV’s work is just to report the readings to the clinicians for interpretation.” (39 y.o. male research assistant, Kenya)

At the same time, they also commented on the value of home-based telehealth for patients and that this actually constituted high-quality care by being patient-centred. As the same research assistant as above commented,

“Being attended at the comfort of your house psychologically relieves one from any form of pressure, and one also feels cared for. It motivates the patient in a way. This is compared to the stress one undergoes all the way to be attended to at the facility due to poor terrain. There is just a way in which appearing at the hospital pisses off most patients. So in one way or the other, home based care is of quality – save for the reasons I had mentioned earlier whereby one is limited in accessing other health services offered at the clinic.” (39 y.o. male research assistant, Kenya)

Both clinicians and CHWs felt they had good coordination with one another when integrating telehealth visits. This reportedly served to increase rapport between participants and CHWs, since when participants saw the relationship between CHW and clinician, they gained trust in the CHW. CHWs planned home visits in advance with participants and clinicians to let them know to expect a call. CHWs reported that clinicians were accessible.

“When you go to the community after finishing everything, you will have to call the clinician so that they can hear from the client. You will have to first explain everything on the ground according to your own assessment that you made. You will take BP measurements and give feedback to the clinician on the findings from the first BP reading all through to the third reading. Then you also give feedback on medication adherence. Is it good or bad? Then, you will also enquire with the client if the adherence was not good. Thereafter, you call the clinician for more consultation. Sometimes, a client may not be ready to disclose everything, but when they talk to the clinician over the phone, they will disclose everything. Sometimes, a client might be adhering well to the drugs, but they do not follow the instructions. When they are conversing with the doctor, they will feel good talking to the doctor while in their comfort zone right at their doorstep. This will motivate them to go the extra mile to explain any other health problems apart from hypertension. At that time the doctor will advise him further by offering referrals to either go to the hospital to see him or go to the nearest place for assistance.” (56 y.o. male CHW, Kenya)

If, for some reason, a CHW had to wait until a clinician was available, they reported using their time on hold to get more info from the participant:

“I have not had any issues because when I am leaving for the field, I inform the providers that I have left and am going to a certain area, and they become aware. If I find them with a client, they will tell me, ‘*Wait a few minutes, I am calling you back*.’ And we work easily. There was no time when they were not available; we communicate well. <… I: *Might they be unavailable because they have other work, and you end up waiting for too long*?> P: No, he will pick up my call and tell me that they have a patient and that they will call after a certain duration. And I will just wait, and that gives me an opportunity to know about other things that were going on within the home that I would have left without knowing.” (40 y.o. female, CHW, Kenya)

In addition to learning about “other things that were going on within the home,” being members of the community gave CHWs opportunities for follow-up that clinic-based providers may not have. For example, CHWs mentioned asking about participants’ health progress during chance meetings outside formal care visits. Additionally, CHWs were able to find participants for clinicians if a patient missed an appointment or if information needed to be relayed to them. “… *most people do not have mobile phones and do not know how to read or write and therefore have to be helped*.” (29 y.o. male Clinical Officer, Kenya)

### Addressing barriers differently

Participants in both arms saw barriers noted that free hypertension medications and elimination of medication stockouts substantially reduced barriers to hypertension treatment (*n.b.,* stockouts were eliminated in both arms of the study). Patient-centred clinic-based care reduced wait times, though community-based care saved additional time and money by eliminating the need to travel to clinic altogether.

## Discussion

Our qualitative study of attitudes towards a telehealth model for hypertension management combined with community-based service provisioning in rural Kenya and Uganda revealed overall positive attitudes and receptivity on the part of intervention participants and providers (inclusive of CHWs, clinicians, and clinic team members). Previously, we have shown that CHW-facilitated hypertension telehealth is both clinically safe and effective, and delivers care at a location preferred by many patients [25]. Participants desired simultaneous involvement and communication between facility-based providers, CHWs, and the participants. While participants felt the facility was a good place to get checked for other illnesses and change medication if necessary, they were open to receiving routine hypertension care in the community. Participants expressed the benefits of going to the facility partly in terms of the personal relationships they valued with their clinical provider and other patients at the clinic. These included the chance to discuss with the clinician concerns that went beyond hypertension and also the possibility of meeting others with the same condition, thereby alleviating feelings of “diagnostic loneliness”.

Despite the benefits of facility-based care, and similar to findings by other researchers, our participants found that the convenience of telehealth promoted patient satisfaction, partly because it is a variation of patient-centred streamlined care [13,24]. Apart from gaining community trust, a key advantage of home-based care was saving time and money that would have been spent on transport and waiting in the clinic, a finding also supported by quantitative surveys [25]. Community-based care also provided an opportunity to educate the community about different illnesses and their treatment options and demonstrate care by bringing services directly to people’s homes. Participants recognised this, and felt community care was good for diagnosing people who were afraid to go to hospital or who did not know their condition, thereby linking them to appropriate care. These benefits, too, expressed the ideal model of care: one delivered in the context of personal relationships, sometimes brokered through social networks and word-of-mouth. This is the heart of patient-centred care.

Such patient-centred, differentiated care delivery (DSD) models have shown promise for HIV management; here we show their promise for hypertension care. Importantly, as other researchers have noted about differentiated care delivery models, “identifying features that are preferred can suggest which are most likely to lead to sustained engagement and retention” [27]. Though participants who did not receive telehealth personally (i.e., control arm participants) worried that CHWs may not have the latest medications, these concerns were not borne out by participants who received the CHW telehealth intervention. Intervention arm participants, in fact, realised that the quality of care was high and that they retained the ability to talk to their clinic-based provider. Further, direct communication between patients, CHWs, and clinicians enhanced trust between patients and their CHWs.

Along these same lines, we also found that trust in telehealth was more tentative among the control group who had not experienced it, compared to those in the intervention group; this mistrust was more focused on concerns about the ability of CHWs to maintain confidentiality than on telehealth data security [13]. In other words, experience of both the CHWs and the way they used telehealth to communicate with clinically-based providers built trust. Other studies, particularly in South Africa, have noted the need to build trust among communities when rolling out telehealth or task-shifting to CHWs [3,9,10,28]. In addition to brokering trust through personal relationships, we found that building awareness must go beyond simply letting people know telehealth is an option and include teaching people how everyone in the process relates to one another and assuring people of the training that the CHWs receive to deliver services in the community [11,12]. For our intervention group, the CHWs succeeded in building these relationships – often pre-existing, as the CHWs were peers and community members – and showing the participants how the moving parts related. Together with the streamlining of care to overcome financial and physical barriers, this social component may partly explain why 48-week hypertension control for our study was significantly higher in the community telehealth arm compared to those randomized to receive hypertension care in the clinic (86% vs 44% with blood pressure <140/90 mmHg; p < 0.001) [25]. Trust, in other words, appeared to be a central feature in promoting the acceptability of our model and sustaining engagement with it; other telehealth interventions could incorporate it into their approach to gaining community approval.

### Limitations

Our study is limited by geography; prior research has shown limited cultural resistance to telehealth in East Africa compared to Western and Central Africa, and thus our findings may not be broadly applicable outside the Eastern and Southern Africa region [11]. However, our finding on the personal-communal nature of how community-based telehealth achieved acceptability may be useful in regions where similar social dynamics are at play in healthcare and health-seeking behaviour. More focused research into the ways people conceive of personalised care, inclusive of biomedical, traditional, and faith-based practices, is warranted, especially in the context of chronic biomedically addressable diseases. Finally, in this pilot study, care location (telehealth versus clinic) was assigned by randomisation rather than patient choice. We are currently studying implementation of hypertension telehealth at scale within a large cluster randomised trial (NCT05768763) where participants can choose their preferred care location in consultation with clinicians to ensure medical appropriateness.

### Recommendations and conclusions

Strategies to foster trust in CHWs and telehealth are important for effective implementation of CHW-facilitated telehealth programs. Further, clinic-based providers should regularly check in with patients to solicit feedback on their experience with telehealth and ensure that patient-provider communication remains intact during check-in visits when patients are not physically attending clinic. Finally, because of the social nature of telehealth as a healthcare system option, incorporating the use of social media and social networks may be one way to raise awareness of this option for hypertension care.

Patients and clinicians found telehealth to be acceptable and to have a similar quality of care to physically attending clinic when used for routine hypertension care. A telehealth model that includes synchronous communication between patients and CHWs in the community with clinicians via telehealth is synergistic, providing clinicians with enhanced insight into patients’ social environment by CHWs, and enhancing patient trust in CHWs and in the quality of care by facilitating direct communication with the clinician. CHW-facilitated telehealth for community-based chronic hypertension care that builds trust through community embeddedness and visible, triadic communication offers one way to improve cardiovascular disease prevention in a culturally appropriate, patient-centred and clinically effective manner in rural Kenya and Uganda.

## Supporting information

S1 File IDI guides: In-depth interview guides. (DOCX)
