# Supplementary material for: Patient perceptions and preferences during a community-based telehealth care model for moderate-to-severe hypertension in rural communities in Kenya and Uganda
Source: PLoS One. 2026 Jul 28;21(7):e0350915. doi: 10.1371/journal.pone.0350915 (PMC13411868; doi:10.1371/journal.pone.0350915)
Supplement: S1 File — (DOCX) [file pone.0350915.s001.docx]

**In-Depth Interview (IDI) Guide 5: Persons with moderate-to-severe hypertension**

**(Revised 15-Aug-2022)**

*After completing informed consent procedures, the interviewer uses the following guide to interview study participants. Interviewer will draw upon the suggested questions and prompts in this guide, but may insert additional follow up questions and probes as needed to explore topics fully. Instructions to interviewers are italicized. Note to interviewer: Ensure privacy and comfort of participant, and enable audio recorder after informed consent and before starting interview. Do not read topic headers aloud. Reiterate consent and confidentiality protections as needed during the interview.*

The purpose of this interview is to find out your opinions about and experiences with accessing health care and treatment services, and specifically your experiences with services provided by health care providers working with the SEARCH-SAPPHIRE Hypertension Treatment study. The information that you share with us will help us to improve services in order to help other people like you. I want to remind you that as we discussed during the informed consent process, the information you share will be kept strictly confidential and will have no effect on the services you receive, so thank you in advance for your open and honest responses.

Today I will ask you questions about your experiences with hypertension care, and about the interactions you had with health care providers. I’ll also ask your opinions about how these hypertension care could be improved. Do you have any questions for me before we start? [*Address questions, start audio recording and begin*.]

**I. Diagnosis and model for hypertension**

1. Can you tell me about when you first learned you had high blood pressure? Where and when did this happen? How did you learn about it? What was your first reaction?
2. How have your feelings about your blood pressure changed over time?
3. Tell me what you understand about high blood pressure?

- What causes it?
- What health problems can high blood pressure cause?
- How likely do you think these problems are to happen to you?

1. Can you think of any other chronic health problems that people you know have? (if participant cannot think of any, suggest diabetes and HIV)
   - What are the consequences of each of these conditions?

- How would you compare high blood pressure to these other conditions in terms of severity of possible consequences?

1. How does your high blood pressure diagnosis affect how you view your role with your family? [By that I mean expectations of yourself for how you are able to provide for your family] How does it affect how others in your family perceive you?
2. How does it affect how you view your role in the community? How does it affect how others in your community perceive you?
3. What do other people in your family say about high blood pressure? What do your friends say about high blood pressure? What do people in the community say about high blood pressure?

**II. Linkage to care and establishing treatment**

1. When you think about the first time you received an explanation about high blood pressure from your healthcare providers – what were you told? What questions did you have? Did you ask your healthcare provider these questions? Were they answered? How did explanations from your health care providers change over time?
   1. How satisfied were you with the quality of the explanations you received from your healthcare provider about your high blood pressure?
   2. How could high blood pressure and treatment for high blood pressure be explained better by your health care team?
2. What is your understanding of the things you can do to help keep your blood pressure controlled? What is your understanding of medical treatments for high blood pressure? What is your opinion about these medical treatments? Probe: how effective are the treatments? How easy are they to take? Please also share with me about alternative treatment options other than medical that you know? [probe for herbal treatments options and their perceptions about it]
3. I would like to learn about your feelings the first time you went to the health facility for high blood pressure treatment.
   1. Probe: Why did you feel that way?
   2. Probe: How have your feelings about going to the health facility changed over time?
4. What are your goals for your high blood pressure? How ready are you to start working towards these goals? (probe: are you already working toward this goal, planning to start working toward this goal, still thinking about whether or not you want to take the first steps toward this goal?) What concerns do you have about your goals or achieving them?
5. What conversations have you had with your healthcare team about goal setting for your high blood pressure?

**III. Maintenance of treatment**

1. What feelings do you have when you think about taking medication for high blood pressure?
2. Tell me what you think about your ability to:
   1. go to the clinic consistently for high blood pressure treatment.
   2. take medications every day for high blood pressure.
3. Tell me about a time when you stopped taking your hypertension medications. What were the reasons that you stopped? Probes:
   1. Health facility issues
   2. Influences in community
   3. Side effects
   4. Mobility
   5. Costs
4. What are some of the challenges you have experienced with hypertension treatment in the past six months (since joining the study)? What else? Probe: What are some of the challenges you have experienced with:
   1. going to the clinic for appointments?
   2. Experiences while at the health facility? (including waiting time, interactions with staff)
   3. Concerns about getting care in the HIV clinic
   4. transportation? Physical mobility?
   5. obtaining medications for your high blood pressure?
   6. taking medications for your high blood pressure every day?
   7. Side effects of medications?
5. Tell me about other chronic conditions that you may have (HIV, diabetes).
   1. [For those with other chronic conditions] What has your experience been getting care for these conditions at the clinic? How are your appointments organized and how do you manage them? How do you feel about getting care for all of these conditions from the same clinic?
6. How do you feel about having hypertension and HIV services delivered in the same clinic?
7. What makes it difficult to remember to go to the clinic? To take your medications?
8. What would make it easier to remember clinic appointments? Going to the clinic?
9. Can you tell me about your interactions with the healthcare providers at the clinic? How about the staff at the clinic?

Please tell me about the things you liked least? What did you like most? (probe for the level of respect during the interactions)

1. What else makes it difficult to go to the clinic for high blood pressure treatment that we haven’t talked about yet?
2. What else makes it difficult to obtain medications that we haven’t talked about yet?
3. What other needs/priorities sometimes get in the way of your high blood pressure treatment?

**IV. Experiences with and acceptability of hypertension care**

1. What do you think about the idea of community-based care for high blood pressure? In what situations is it better to get care at the clinic? In what situations is it better to get care at home?
2. How does community-based care for high blood pressure make it easier to receive high blood pressure treatment?
3. How does community-based care for high blood pressure make it harder to receive high blood pressure treatment?
4. What concerns do you have about community-based care for high blood pressure?

**V. Recommendations to improve hypertension care**

Now I’d like to ask your recommendations about how the hypertension care you have been receiving could be improved.

1. What are the main things that could make it easier to access hypertension care, if anything?
2. What would you change about the way you are currently receiving hypertension care?
3. What would you change about the way you are currently receiving medical care for other issues besides hypertension?
4. Please share anything else with me that you would like our study team to know.

*THANK STUDY PARTICIPANT FOR THEIR TIME AND END INTERVIEW.*

**[DCT] In-Depth Interview (IDI) Guide 7: DCT Hypertension CHVs/VHTs**

**(Revised 2023-02-17)**

*Interviewer: After completing informed consent procedures, the interviewer uses the following guide to interview the CHVs/VHTs supporting community-based hypertension monitoring and treatment. Interviewer will draw upon the suggested questions and prompts in this guide, but may insert additional follow up questions and probes as needed to explore topics fully. Instructions to interviewers are italicized. Note to interviewer: Ensure privacy and comfort and enable audio recorder after informed consent and before starting interview. Do not read topic headers aloud. Reiterate consent and confidentiality protections as needed during the interview.*

Thank you again for meeting with me today. As we discussed, we are conducting interviews with the CHVs/VHTs to understand your experiences with and perceptions of the SEARCH-SAPPHIRE hypertension treatment services provided in this community. Your participation is very important because we want to learn about your experiences interacting with intervention participants to provide community-based hypertension care. I may make some notes as we talk in order for me to remember questions that I want to ask you later. Also, I will record the discussion so that we can analyze what was said later.

**I. CHV/VHT workflow**

1. Can you tell me about your current responsibilities as a CHV/VHT?
   1. Which of your responsibilities do you think are the most impactful for the health of your community?
   2. With so many pressing concerns, I’m interested in learning how you determine priorities as a CHV/VHT.
      1. Probe: where does high blood pressure treatment fit in?
2. Now I want to talk about your experience treating high blood pressure in your community
   1. What is easy about treating high blood pressure as a CHV/VHT?
   2. What are the main challenges you have experienced?
3. Can you tell me about the training you have received so far on measuring blood pressure and supporting medication adherence for hypertension?
   1. What was useful about this training?
   2. What additional training would be helpful?
4. What are some of the common health problems caused by high blood pressure that you have seen in your community?
   1. How likely do you think these problems are to happen to people in your community who have high blood pressure?
   2. How would you compare high blood pressure to other conditions in terms of severity of possible consequences in your community?

**II. Experience with the community-based hypertension intervention**

1. What do you think about the idea of community-based hypertension care?
   1. Tell me about the patients for whom you think it was useful.
      1. Probe: Can you tell me a story about one of them?
   2. Tell me about the patients for whom it was not useful or did not work well.
      1. Probe: Can you tell me a story about one of them?
2. How does the quality of care differ between clinic-based and community-based (with clinician phone consultation) hypertension care?
3. What issues have you experienced with contacting clinicians to conduct phone-based hypertension care assessment? Are they ever not available and, if so, what do you do?
4. What concerns do you have about community-based hypertension care?
5. What elements of community-based hypertension care do you think were most impactful on the trial participants?

**II. Recommendations to improve the community-based hypertension intervention**

1. In what ways can the community-based hypertension care model be improved to make hypertension care easier for patients?
2. In what ways can the community-based hypertension care model be improved to make hypertension care easier for CHVs/VHTs?
3. What other types of services do you think can be integrated with community-based hypertension care? What tools would you need to add these services?
4. Please share anything else with me that you would like our study team to know.

**[DCT] In-Depth Interview (IDI) Guide 7: DCT Hypertension Clinicians**

*Interviewer: After completing informed consent procedures, the interviewer uses the following guide to interview the clinicians providing hypertension treatment. Interviewer will draw upon the suggested questions and prompts in this guide, but may insert additional follow up questions and probes as needed to explore topics fully. Instructions to interviewers are italicized. Note to interviewer: Ensure privacy and comfort and enable audio recorder after informed consent and before starting interview. Do not read topic headers aloud. Reiterate consent and confidentiality protections as needed during the interview.*

Thank you again for meeting with me today. As we discussed, we are conducting interviews with clinicians to understand your experiences with and perceptions of the SEARCH-SAPPHIRE hypertension treatment services in your clinic and community. Your participation is very important because we want to learn about your experiences interacting with intervention participants to provide hypertension care, either in the clinic or over the phone during a community-based hypertension care visit. I may make some notes as we talk in order for me to remember questions that I want to ask you later. Also, I will record the discussion so that we can analyze what was said later.

**I. Screening/Diagnosis**

1. When you think about measuring blood pressure in your clinic:
   1. how important does this seem compared to other priorities that you have?
   2. how feasible does this feel to you?
   3. How does this idea make you feel when you weigh blood pressure screening with your many other priorities?
2. If you diagnose someone with hypertension, how likely is that to have a positive impact on their health?
3. Based on your current training, how confident are you that you have the knowledge and skills needed to diagnose someone with hypertension? What additional training would be helpful for improving your confidence?
4. Based on current equipment and staff available in your clinic, how capable are you of screening for hypertension?
5. Based on current protocols and standard work in your clinic, how capable is your clinic of incorporating hypertension screening into standard clinic workflow? Where would hypertension screening ideally occur in your clinic?
6. What are some of the other drawbacks to being able to offer effective hypertension screening? (probe; at the facility, patient and community/family level)
7. What health problems can high blood pressure cause? How likely do you think these problems are to happen to your patients? How would you compare high blood pressure to other conditions in terms of severity of possible consequences?

**II. Treatment**

1. When you think about treating hypertension in your clinic:
   1. how important does this seem compared to other priorities that you have?
   2. how feasible does this feel to you?
   3. How does this idea make you feel when you weigh hypertension treatment with your many other priorities?
2. What are some of the challenges you experience with:
   1. Maintaining drug supply
   2. Maintaining functioning blood pressure cuffs
   3. Incorporating hypertension treatment into your workflow
   4. Ensuring patients come back for follow-up appointments
   5. Managing complications of hypertension
   6. Referring patients to a higher level of care when needed
3. What would make it easier to manage hypertension in your setting?
4. Tell me what you think about your ability to:
   1. Manage routine hypertension
   2. Manage complicated hypertension or complications of hypertension (e.g. kidney failure, heart failure)
5. Thinking about difficulties that patients face in your clinic:
   1. What is the most challenging barrier patients face to consistently achieving hypertension control
   2. What would make it easier for patients to consistently achieve hypertension control
6. What else makes it difficult to treat hypertension that we haven’t talked about yet?

**III. Experience with clinic- and community-based hypertension care**

1. What do you think about the idea of community-based hypertension care? Tell me about the patients for whom you think it was useful. Tell me about the patients for whom it was not useful or did not work well.
2. How does the quality of care differ between clinic-based and community-based (with clinician phone consultation) hypertension care?
3. How do phone visits for community-based hypertension care fit into your workflow? What are some of the challenges you have experienced?
4. What concerns do you have about community-based hypertension care?
5. Where there times where you weren’t able to get enough clinical information during a phone visit or where you had to ask a participant to come to the clinic? Tell me about those experiences. What would have made these experiences easier?
6. What elements of community-based hypertension care do you think were most impactful on the trial participants?

**IV. Recommendations to improve the community-based hypertension intervention**

1. In what ways can the *community*-*based* hypertension care model be improved to make hypertension care easier for *patients*? In what ways can *clinic-based* hypertension care be improved to make hypertension care easier for *patients*?
2. In what ways can the *community-based* hypertension care model be improved to make it easier for *clinicians* to provide hypertension care? In what ways can *clinic-based* hypertension care be improved to make it easier for *clinicians* to provide hypertension care?
3. What other types of services do you think can be integrated with community-based hypertension care? What tools would be needed to add these services?
4. Please share anything else with me that you would like our study team to know.
