# Supplementary material for: Patient perceptions and preferences during a community-based telehealth care model for moderate-to-severe hypertension in rural communities in Kenya and Uganda
Source: PLoS One. 2026 Jul 28;21(7):e0350915. doi: 10.1371/journal.pone.0350915 (PMC13411868; doi:10.1371/journal.pone.0350915)
Supplement: Attachment — Submitted filename: Letter to Editors and Reviewers RnR2_2026-05-08.docx [file pone.0350915.s004.docx]

2026-05-08

PONE-D-26-01917R1

Patient perceptions and preferences during a community-based telehealth care model for moderate-to-severe hypertension in rural communities in Kenya and Uganda

Dear editors,

Per the response of Reviewer 1 to question 5, indicating that the manuscript is not presented in intelligible English, we have no undertaken a thorough review of the article’s grammar and spelling.

We only found a few instances of verbs needing to be changed (e.g. singular to plural), one instance of a word being duplicated, and a few missing commas. We have not felt that a tracked changes document would be necessary at this point. If you wish us to create one, please let us know.

Best regards,

Jason Johnson-Peretz
