# Supplementary material for: Patient perceptions and preferences during a community-based telehealth care model for moderate-to-severe hypertension in rural communities in Kenya and Uganda
Source: PLoS One. 2026 Jul 28;21(7):e0350915. doi: 10.1371/journal.pone.0350915 (PMC13411868; doi:10.1371/journal.pone.0350915)
Supplement: Attachment — Submitted filename: Letter of Response to Editors and Reviewers 2026-04-08.docx [file pone.0350915.s003.docx]

**Letter of Response to Editors and Reviewers**

**Manuscript ID:** PONE-D-26-01917

**Manuscript Title (previous):** The acceptability of a community-based telehealth care model for moderate-to-severe hypertension in rural communities in Kenya and Uganda

**Dear editors,**

**Thank you for the opportunity to address the helpful comments provided by the reviewers. We have edited the manuscript in response to these comments. We provide both a clean and marked copy of the revised manuscript with this resubmission, and we directly quote the changes in our responses below. We have done our best to undertake a thorough and responsive revision of the manuscript. We hope that the editors and reviewers agree that the revised manuscript is stronger as a result of these very helpful critiques.**

**Below please find each comment with our response indented and in bold print:**

*Editorial Comments to Author:*

1. Please ensure that your manuscript meets PLOS ONE's style requirements, including those for file naming. The PLOS ONE style templates can be found at

[https://journals.plos.org/plosone/s/file?id=wjVg/PLOSOne_formatting_sample_main_body.pdf](https://urldefense.com/v3/__https:/track.editorialmanager.com/CL0/https:*2F*2Fjournals.plos.org*2Fplosone*2Fs*2Ffile*3Fid=wjVg*2FPLOSOne_formatting_sample_main_body.pdf/1/010f019d41aaf7ca-dd2eced5-5ee1-4151-b431-c4d19ca7d6e3-000000/MqG5VGy5CtfF9LSXxFsoP-fwV8vMJY94myC9CHPr7aM=256__;JSUlJSUlJQ!!LQC6Cpwp!sEyb5mSfNRZZmSiApQiHp2G5knCqHG2CzCxfLUxs6d0nxqBbFrao5MVQkRvUhKmEvBAN1uQeVt6lfxi3aRORm5s$) and

[https://journals.plos.org/plosone/s/file?id=ba62/PLOSOne_formatting_sample_title_authors_affiliations.pdf](https://urldefense.com/v3/__https:/track.editorialmanager.com/CL0/https:*2F*2Fjournals.plos.org*2Fplosone*2Fs*2Ffile*3Fid=ba62*2FPLOSOne_formatting_sample_title_authors_affiliations.pdf/1/010f019d41aaf7ca-dd2eced5-5ee1-4151-b431-c4d19ca7d6e3-000000/fN57hFBG5x8vl3f5S5UIdxAgrxB0Mmxk4_Oke_wix7g=256__;JSUlJSUlJQ!!LQC6Cpwp!sEyb5mSfNRZZmSiApQiHp2G5knCqHG2CzCxfLUxs6d0nxqBbFrao5MVQkRvUhKmEvBAN1uQeVt6lfxi3r3HKXi4$)

**We have made this change**

2. Please include a complete copy of PLOS’ questionnaire on inclusivity in global research in your revised manuscript.

**We have included a copy of this questionnaire. Two-thirds of the study co-authors are based locally in the countries where the research was conducted, including the co-senior author.**

3. Thank you for stating the following financial disclosure:

“The study was supported by grants from the National Institutes of Health (U01-AI150510 Multiple-PI award to DVH, MLP, MRK; K23HL162578 to MDH). The content is solely the responsibility of the authors and does not necessarily represent the official views of the NIH.”

Please state what role the funders took in the study.  If the funders had no role, please state: "The funders had no role in study design, data collection and analysis, decision to publish, or preparation of the manuscript."

Please include this amended Role of Funder statement in your cover letter; we will change the online submission form on your behalf.

**The funders had no role in study design, data collection and analysis, decision to publish, or preparation of the manuscript.**

4. Please note that funding information should not appear in any section or other areas of your manuscript. We will only publish funding information present in the Funding Statement section of the online submission form. Please remove any funding-related text from the manuscript.

**We have now removed the funding information from the manuscript.**

5. You have indicated that data is available from “[Douglas.Black@ucsf.edu](mailto:Douglas.Black@ucsf.edu)” .Please can we ask you to provide us with a general contact email address for the data requests, so readers can request access in perpetuity. If a general email is not available please provide a link to a website where readers can obtain access to data.

**We have now changed this to: “Please direct further inquiries to the SEARCH Scientific Review Committee via** [**SEARCHProjectInfo@gmail.com**](mailto:SEARCHProjectInfo@gmail.com) **.”**

6. Your ethics statement should only appear in the Methods section of your manuscript. If your ethics statement is written in any section besides the Methods, please delete it from any other section.

**We have now moved the ethics statement to the methods section of the manuscript.**

7. Please include captions for your Supporting Information files at the end of your manuscript, and update any in-text citations to match accordingly. Please see our Supporting Information guidelines for more information: [http://journals.plos.org/plosone/s/supporting-information](https://urldefense.com/v3/__http:/track.editorialmanager.com/CL0/http:*2F*2Fjournals.plos.org*2Fplosone*2Fs*2Fsupporting-information/1/010f019d41aaf7ca-dd2eced5-5ee1-4151-b431-c4d19ca7d6e3-000000/jrDVXgHXxdc1CHsxNo62EJspIxKOUTv1aaZkGYCMpxw=256__;JSUlJSU!!LQC6Cpwp!sEyb5mSfNRZZmSiApQiHp2G5knCqHG2CzCxfLUxs6d0nxqBbFrao5MVQkRvUhKmEvBAN1uQeVt6lfxi3ngqvTNA$).

**We now include supporting information files with the file names:**

**S1_HTN-S IDI Guides 2023-02-17**

**S2_Figure 1**

*Reviewer 1 Comments to the Author*

The study evaluates “acceptability,” but does not explicitly anchor findings in a formal implementation science framework such as:
• Theoretical Framework of Acceptability (TFA)
• CFIR (Consolidated Framework for Implementation Research)
• RE-AIM
• DSD (Differentiated Service Delivery) frameworks

The current framing is descriptive rather than conceptual.
Suggestion:
Add a short conceptual paragraph clarifying:
• How “acceptability” is defined (affective attitude? perceived effectiveness? trust? burden?)
• Whether analysis was mapped to any implementation construct
• How findings inform implementation science beyond this context

This would significantly increase scientific depth and generalizability.

**We have made several changes to address these concerns. First, we have altered the title to match more closely the intention of the paper: “Patient perceptions and preferences during a community-based telehealth care model for moderate-to-severe hypertension in rural communities in Kenya and Uganda”.

Second, in the background section, we added the final sentence, “We sought to assess people’s experiences with each model (community vs clinical care), including their preferences and perceptions about quality of care. “From this comparison of models, we derived evidence for both the general acceptability of the telehealth model and participant-perceived limits to its utility (i.e. the range of acceptability).”**

**Third, in the methods section, we added the following: “In line with the qualitative tradition, we used open-ended questions to elicit feelings, beliefs, and perceptions about the intervention (whether positive or negative), and a semi-structured format to delve into the reasons for those feelings. This allowed us to uncover a broad range of information not captured in quantitatively-focused surveys that deploy *a priori* conceptual categories of acceptance or rejection of an intervention component. We have included the interview guides as supplementary material for reference; the pertinent questions appear in Table 2.”**

**We note that most of the frameworks Reviewer 1 cited are quantitative in orientation. Although the overall study used CFIR to map implementation context, for this paper we took an inductive, interpretive approach, even as we did keep in mind implementation science frameworks more broadly. Primarily following the interpretivist tradition meant taking a bottom-up approach to see what the data tell us, rather than a more top-down approach using *a priori* constructs provided in frameworks like CFIR.**

**To illustrate this difference more clearly, we have therefore added a table 2 with the question stems we used to ascertain acceptability. Although the data collection instruments were informed by IS constructs, including acceptability, the questions were kept open-ended to elicit information from participants that these constructs may not have captured in this context and among these communities. We chose to deploy such open-ended questions to elicit feelings, beliefs, perceptions about the intervention, whether positive or negative, and to delve into the reasons or backgrounds to those feelings.**

**For further clarification, we want to be sure the reviewer understands our aim in this paper is not to inform IS frameworks and methods, but rather to present the underlying cultural dynamics that have implications for medical practice and policy and related public health interventions. We hope this difference is seen as complementary, rather than competitive.**

Also clarify
How participants were selected (maximum variation? based on BP control? adherence?)
• Whether data saturation was achieved
• Whether sampling differed by country, sex, or outcome
• Whether non-adherent or dissatisfied participants were deliberately included

**Thank you for these suggestions. In the methods qualitative subsection, we now clarify the following: “For the qualitative sub-study, a gender-balanced team (3 men, 2 women) of trained qualitative researchers conducted audio-recorded, in-depth, semi-structured interviews with purposively selected providers (N=15) and participants (N=40) from the main trial between 25 January and 28 April 2023 (Table 1); participants were balanced by sex, community, blood pressure status at 6 months (elevated or controlled), and HIV status, but selection was agnostic to intervention satisfaction as our intention was to uncover degrees of satisfaction through a random selection within these purposive categories. Clinicians were all study staff. We achieved data saturation with these numbers of participants.”**

Also, Expand mechanistic interpretation: Link themes (trust, triadic communication, CHW embeddedness) to outcome improvement pathways.

**We now try to make this more clear in the conclusion by changing the tense and adding a more explicit causal chain: “A telehealth model that includes synchronous communication between patients and CHWs in the community with clinicians via telehealth is synergistic, providing enhanced insight into patients’ social environment by CHWs and enhancing trust in CHWs and in the quality of care by facilitating direct communication with the clinician. CHW-facilitated telehealth for community-based chronic hypertension care that builds trust through community embeddedness and visible, triadic communication offers one way improve cardiovascular disease prevention in a culturally appropriate, patient-centred and clinically effective manner in rural Kenya and Uganda.”**

Also, Some repetition in discussion (trust theme).

**We appreciate this observation and note that we sought to compare the role of trust (that we uncovered in our findings) for both intervention and control groups, hence the two-part repetition in the discussion: one considered trust from the intervention group, and the other triangulated its importance based on findings from the control group.**

Some quotes are long and could be shortened.

**Following the interpretivist anthropological tradition established by Clifford Geertz, we have retained the length of the quotes in order to achieve 'thick description'. We have chosen this so that other social scientists are able to evaluate the data for more ethnographically-oriented purposes. For a general overview of the concept of thick description, see Ponterotto (2006). “Brief Note on the Origins, Evolution, and Meaning of the Qualitative Research Concept ‘Thick Description’”. *The Qualitative Report*, vol 11, No 3, pp538-549. For Geertz, see: Geertz (1973). *The Interpretation of cultures: Selected Essays*. NY: Basic Books.**

Clarify whether medication stock-outs were eliminated in both arms.

**Thank you for this suggestion. We now clarify this in a parenthetical note in the results section for the readers’ benefit: “Participants in both arms saw barriers noted that free hypertension medications and elimination of medication stockouts substantially reduced barriers to hypertension treatment (*n.b.* stockouts were eliminated in both arms of the study).”**

Clarify if clinicians were Ministry staff or study staff.

**As noted above, we now clarify this in the methods section: “…Clinicians were all study staff. We achieved data saturation with these numbers of participants”**

Consider adding a figure illustrating the telehealth workflow.

**Thank you for this suggestion. We have now added a figure 1 illustrating the telehealth workflow.**

*Reviewer 2 Comments to the Author*

The qualitative part of the methods has been written well covering the plan of analysis too*.*

A flow diagram of the study is missing that further delineates intervention and control groups. This will help the audience to dissect the overall flow of the study.

**Thank you for this suggestion. We have now included a reference to the main quantitative paper that does include such a consort diagramme. Such a diagramme, apart from repeating already published information, seems out of place in this paper that reports findings from the qualitative sub-study with a purposively selected sample of participants and providers.**

A little expansion on the methods of the overall study is needed here in the qualitative aspect of the project as well. I know this has been provided in quantitative part seperately, just to make it more understandable for the readers.

**Thank you for this suggestion. We are uncertain what exactly is being requested, however – do you intend that we repeat portions from the overall study context subsection again in the qualitative subsection?

We do expand a little on the qualitative sampling categories as follows, and we hope this addresses the reviewer’s concern: “with purposively selected providers (N=15) and participants (N=40) from the main trial between 25 January and 28 April 2023 (Table 1); participants were balanced by sex, community, age, and HIV status, but selection was agnostic to intervention satisfaction as our intention was to uncover degrees of satisfaction through a random selection within these purposive categories. The number selected achieved data saturation.”**

**The authors in the limitations haven't mentioned about contamination bias, s**ince the control group participants might receive the intervention and vice-versa, kindly shed some light on this aspect as well.

**Thank you for this comment. In the main trial, we had no instances where control arm participants received the intervention. Intervention arm participants could receive care in the clinic if recommended by the clinician. Here is what we stated in the main paper (the PLOS Medicine article we cite as reference 25): “In the telehealth group, 95% of post-baseline visits were conducted at home by telehealth (485/512), and only 5% of visits were conducted in the clinic due to clinician recommendation (27/512). All post-baseline visits in the clinic-based arm occurred in the clinic (373/373).”**

How about the lost to follow-up data?

**Although this suggestion appears to relate more to the main (quantitative) paper, rather than to this purposively selected qualitative sample, we now specify in the methods section: “Loss to follow-up was minimal, though slightly higher in the control (clinic-based) arm (3 vs 9 participants).”**

How about the duration of the intervention?, methodological details are missing.

**Thank you for pointing out this oversight. We now specify in the methods section that: “The intervention lasted 48 weeks.”**

**Again, we appreciate the thoughtful and helpful comments provided by the reviewers and editor. Thank you for the opportunity to have our manuscript considered for publication in PLoS One.**

**Sincerely,**

**Jason Johnson-Peretz**
